# The influence of transformations in supply on methamphetamine initiation among people injecting opioids in the United States

**DOI:** 10.1186/s12954-024-00976-1

**Published:** 2024-03-05

**Authors:** Sarah Mars, Jeff Ondocsin, Nicole Holm, Daniel Ciccarone

**Affiliations:** University of California, San Francisco, USA

**Keywords:** Drug supply, Methamphetamine, Opioids, Injecting drug use, Drug initiation , Drug trajectories

## Abstract

**Background:**

Co-use of methamphetamine (MA) and opioids (pharmaceutical pills, heroin and fentanyls) has increased in the United States and is represented in rising mortality. Although coinciding with the import of low cost, high potency and purity methamphetamine, the relationship between supply and demand in propelling this polydrug use is not well understood. We consider the influence of macro changes in supply on the uptake of opioid and methamphetamine co-use by injection at the level of individual drug and injection initiation in West Virginia, a state which leads the US in drug overdose mortality.

**Method:**

We recruited n = 30 people for semi-structured interviews who self-reported injecting heroin/fentanyl and using methamphetamine by any route at a West Virginia syringe service program and through snowball sampling. Interviews were recorded and transcripts analyzed using a thematic approach. Ethnographic observation was also conducted and recorded in fieldnotes. Sequence of substance and mode of use initiation and use trajectories for opioids and stimulants were charted for each participant.

**Results:**

A clear pattern of individual drug initiation emerged that matched each successive supply wave of the US overdose epidemic: 25 participants had initiated opioid use with pills, followed by heroin, often mixed with/replaced by fentanyl, and subsequently added methamphetamine use. For participants, the supply and consumption of opioid analgesics had set in motion a series of steps leading to the addition of stimulant injection to existing opioid injecting repertoires. Unlike other studies that have found a birth cohort effect in patterns of initiation, participants showed the same sequence across age groups. Considerations of economy, availability, dependence, tolerance and the erosion of taboos that marked transitions from opioid pills to heroin injection influenced these subsequent trajectories in novel ways. The form, timing and extent of opioid and stimulant consumption was influenced by four stages of the changing drug supply, which in turn reflected back on demand.

**Conclusion:**

Transformations in the social meaning and supply of methamphetamine enabled these transitions while other desired, non-injectable drugs were difficult to obtain. We discuss policy implications of injectable drugs’ market dominance at this location and possible interventions.

## Background

### Macro and micro influences on drug initiation

In this paper we examine the uptake of methamphetamine (MA) by injection among people using opioids in West Virginia, United States (US). Through this we consider the relationship between individual initiation into particular substances by injection and changes in the drug supply. Historically sudden increases in use, or epidemics, have often occurred when major supply side changes in availability and price or technological innovations, such as the hypodermic syringe, meet new or existing demand [1, 2].

How individuals’ life contexts and events intersect with the macro-level dynamics of drug epidemics adds to this complexity. At an individual level, genetic factors can play a part, acting differently on initiation and the development of heavier use, although findings regarding their contribution to each have been inconsistent and vary by substance [3, 4]. ‘Environment’, a vast category of influences, encompasses socio-economic settings, cultural framings of drug use, geographical location, personal life experiences, social networks, legal frameworks and the availability of therapeutic interventions to cease or modify use. Supply, the availability, quality and price of substances at any given time, is also a part of this environment.

These epidemics have usually been identified by a single substance, whether amphetamine in post-Second World War Japan or heroin in 1980s Britain [5, 6]. The current US overdose epidemic, by contrast, is defined by outcome: an ongoing increase in deaths from multiple substances. US overdose mortality from any drug started to rise in 1979 [7] and then more steeply from the late 1990s when a series of opioids and later stimulants were implicated. Unintentional drug overdose mortality since 1999 has now reached almost one million, three quarters of which involved an opioid [8, 9], accumulating in four interrelated ‘waves’ [10–12].

### Supply and demand drivers of drug initiation and transitions

During the first three overdose waves of opioid pills, heroin and fentanyl, the supply and demand mechanisms underpinning initiation and transition between drugs and modes of use varied: first, the prescription and diversion of a new pharmaceutical opioid supply, less stigmatized than heroin, drew in new initiates. The consumption of opioid pills then functioned for some as a true ‘gateway’ to heroin use and from there to injecting driven by both supply and demand: increasing dependence, intensified drug tolerance and opioid pill shortages resulting from pharmaceutical reformulation and prescribing changes drew people to heroin as a more available cost-effective alternative [13–15]. Availability of low cost, high purity heroin was high at this time following changes in sourcing [16]. Shifting involvement by those consuming opioid pills in peer groups and social contexts engaged in heroin use and injecting helped to erode previously held taboos against these activities [13, 17, 18]. At the same time, new opioid naïve consumers were also drawn into heroin use. Birth cohort data show that individuals born before 1980 were more likely to initiate opioids with heroin while those born after more frequently initiated with non-medical use of prescription opioids [19]. By 2015, however, new opioid initiates were more frequently starting with heroin once again.

Subsequent transition from heroin to fentanyls or to combinations of both in the third wave was supply-led through the covert adulteration and substitution of the drugs sold as ‘heroin’ or as counterfeit pharmaceuticals [20–22], i.e. although some people expressed a preference for fentanyl over heroin, the absence of overt fentanyl sales impeded real choice [23, 24]. In recent years there has been increasing evidence of fentanyl being identified as such at the point of sale, thus making a transition from heroin to fentanyl more distinct [25, 26]. However, regional variation persists and fentanyl concealment continues in the form of counterfeit pills. For individuals, progression along this path is not continuous or inevitable as new initiates moved in and out of use and others who had stopped return.

Researchers have posited that the adulteration and increasing replacement of the heroin market with fentanyl was supply-imposed due to heroin ‘supply shocks’, possibly intensified by increased demand from an expanding heroin using population [23]. Following the introduction of fentanyl, its advantages to suppliers in terms of profit likely sustained its presence in the market. By dose-equivalency, fentanyl is considerably cheaper to manufacture than heroin [27], offers a lower risk of detection during trafficking due the smaller volumes required and is invulnerable to the hazards of crop cultivation [23].

Mexico and China remain the two primary sources of fentanyl to the US, with fentanyl arriving via international mail or trafficked across the US-Mexico border in the form of powder or counterfeit, pressed pills. Fentanyl from China typically has purity greater than 90% compared to fentanyl from Mexico, averaging less than 10% [28]. However, strict regulations on precursor chemicals imposed by China in 2018 opened the door to a supply of fentanyl and its precursor chemicals from India. Despite subsequently enacting restrictions similar to those taken by China, India’s enforcement difficulties have allowed the supply to continue and may promote further diversification of the fentanyl precursor market [29, 30]. Since 2021 the weight of fentanyl seized annually by US Customs has more than doubled [31].

### The fourth wave: opioids and stimulants

During these changes in opioid supplies and use, there has been an increase in the co-use of MA on top of existing opioid usage [10, 32–34]. On the demand side, research to date shows that those adding MA to heroin/fentanyl use during this fourth wave of the US overdose epidemic have favored it for a range of reported effects: to prevent or reverse opioid overdose [35]; to minimize symptoms of opioid withdrawal and craving [36]; to detox from heroin or minimize its use [37]; to balance the sedative effects of opioids and to enhance the effects of the opioid high [37–42]. Our study’s findings on demand influences are explained in greater detail in a companion paper [41].

Co-use or combined use of MA with heroin as a ‘goofball’ has not always been popular [43] and the primary ingredients available have also changed significantly in the last decade. Where fentanyl has replaced or adulterated heroin, this represents a 30–40-fold increase in potency at the mu receptor and in the case of some fentanyl analogs, much higher potency [44]. Since 2011 the MA seized in the US has reached historically high levels of potency and purity exceeding 90% [45, 46].

### Methamphetamine supplies

After the introduction of controls on precursor chemicals in the US and Mexico [47], smaller scale, less detectable production methods diffused westwards into rural areas of the US, including West Virginia [46, 48]. The ‘one-pot’ production method is the most recent, known as ‘shake ‘n’ bake’ for its use of a shaken plastic bottle. It requires small quantities of pseudoephedrine or other chemicals but results in a low quality, low yield product [46]. However, manufacturers in Mexico outpaced domestic output using variants of phenyl-2-propanone (P2P)-based manufacturing method which facilitates larger scale, higher purity production [28, 49]. By 2010, most of the MA available in US was produced using this P2P method [46, 50]. Since 2014 the availability of MA has risen steeply in the US, with the number of seized samples increasing 75% from 2014 to 2019, and the increase in MA and opioid co-use witnessed in the overdose epidemic’s fourth wave coincides with these supply changes [46, 51]. However the relationship between supply and demand in propelling this co-use, its modes of administration and sequences of initiation are not fully understood.

### Supply influences on modes of administration

For both MA and opioids, modes of administration have been linked to the product’s country of origin and its availability [52]. Smoking has been associated with Mexican-sourced MA while injecting, snorting and eating are more commonly found with use of the domestic US product [49]. Cunningham et al. showed that, between 1992 and 2004, the prevalence of these modes of use shifted with each country’s production levels [49]. However, it is unknown whether these patterns of use involved individuals switching between modes of administration and source-types or initiating first time use. In national population surveys, MA is most commonly used by non-injection routes such as smoking or snorting [53]. Powder heroin, originally sourced from Colombia and later Mexico, sold in the eastern US, can be snorted and dissolves easily for injecting but is not suitable for smoking [54]. Conversely, Mexican-sourced black tar heroin can be smoked or injected after heating [55].

### Sequence of drug initiation and supply

Studies of the sequence of drug initiation and its possible causative links have focused particularly on the so-called ‘gateway’ effect where the use of drugs legally available to adults such as tobacco, alcohol and cannabis, precedes and, it is argued, increases the likelihood of subsequent opioid, cocaine and MA use [56–59]. However, few studies have reported on the patterns of initiation between subsequent ‘hard’ drugs, for instance whether MA use tends to precede or follow heroin uptake or vice versa, and what role the market plays in these transitions. In contrast, Golub et al. advance the ‘drug generations’ model where, typically, people in their late teens initiate drug use with popular substances of the time, giving rise to episodic epidemics. These specific drug epidemics then plateau and fall as new generations reject once popular but now discredited substances and choose alternatives. These epidemics start in small subpopulations and then expand to a broader population [60].

Our research focus is on people who inject drugs in southwestern West Virginia where the overdose mortality rate has been among the highest in the nation since 2013 [61] with those involving MA climbing steeply from 2015 [62]. The state experienced some of the most intense and sustained opioid pill prescribing in the US followed by the introduction of previously rare heroin and then fentanyl [63]. These supplies were made available amidst extreme income inequality, high unemployment and a job market that relied heavily on manual labor in high risk occupations where accidents and injuries were common [64, 65]. The study location has a high rate of poverty and was experiencing an injection-related HIV outbreak at the time of the research [66]. In this paper we consider the connections between the macro supply changes of the US overdose epidemic and individual drug use trajectories; we focus particularly on how supply has influenced a series of steps leading to the uptake of methamphetamine and opioid co-use by injection from the experiences of those involved. To our knowledge, it is the first paper to do so.

## Methods

In 2017 the study team conducted primary fieldwork on fentanyl perceptions and use adaptations in West Virginia. Subsequently, study personnel maintained contact with key informants in the area and returned in September 2019 to Huntington, WV to investigate the emerging co-use of stimulants and opioids during the fourth wave of the opioid crisis. The research project uses rapid ethnography to investigate novel methods, forms and changing contexts of heroin use around the country [67]. Rapid ethnography can quickly produce detailed knowledge about emerging health problems and identify areas for further research. This method, like traditional ethnography, builds findings primarily around the lived experience and observations of the research participants, in this case around substance use and its health and social sequelae. Fluctuations in the drug supply can occur rapidly, and changing patterns of use may influence health outcomes in ways that are not easily captured or predicted by survey methodology or traditional ethnography.

### Recruitment and sample

For one week, five investigators conducted in-depth semi-structured interviews, asking questions about drug use history and progression, current substances used, injection initiation and practices, drug testing methodology or suspected contamination, concerns around HIV or HCV, experiences of overdose, and other related topics. Interviews lasted 30–60 min, were audio-recorded, and participants were compensated $20. Participants (n = 30) were aged 18 or older and self-identified as injecting heroin/fentanyl and using methamphetamine by any route.

This was a non-random convenience sample in which primary recruitment occurred at a syringe services program in Huntington, West Virginia with some snowball sampling from among acquaintances, friends and partners of research participants who did not directly attend the syringe services program but were introduced to the research team and formally recruited later. The research team initially approached clients attending the syringe exchange and informed them of the research; formal consent was provided orally to maintain confidentiality. Interviews were conducted in private rooms at the exchange site, in quiet locations on the outside grounds of the site and at participant’s homes or living spaces. Recruitment criteria excluded anyone intoxicated or otherwise unable to consent but no instances arose. Participants included 18 cis-gender men and 12 cis-gender women, ranging in age from 24 to 49. One participant identified as white and Native American and 29 identified as white.

The research team also collected ethnographic observations of substance preparation and use (n = 6), practices around injection and other forms of drug consumption, housing and living conditions and relevant aspects of the built environment participants noted on tours of drug using and selling areas. Team members asked participants with whom they had particular rapport if they would be interested in providing tours of drug using and selling areas, if applicable. A few participants directly invited ethnographers to observe their substance use before being asked by the team. These observations were informal, lasting for up to a half day, although some participants spent more time than this over multiple visits. Most participants were interviewed prior to allowing ethnographers to observe substance use and preparation. These observations were recorded in brief *in-situ* notes that were expanded upon in daily collaborative fieldnote writing, while drug preparation and consumption was recorded through photo and video. The team produced daily fieldnotes, which informed the refinement of the interview guide for the rest of the week in an iterative process—e.g., questions about local versus out-of-town dealers and the drug barter economy were added based on early interviews. We have changed all names of quoted participants to protect their privacy. A Federal Certificate of Confidentiality protects the data and the University of California, San Francisco Human Research Protection Program approved the study.

### Analysis

Audio recordings of interviews were professionally transcribed, corrected and read through by the authors who drafted analytic memos for each transcript according to the methods used by Christopoulos et al. [68] and compiled them into a single searchable table using MS Word. The interview guide informed the early development of the analytic memo structure, while a constant-comparative methodology allowed for the emergence of themes from close reading and text comparison across data sources [69]. The authors met weekly to discuss and refine emergent themes. Memos addressing overarching themes were also prepared. We compiled an Excel spreadsheet to chart the order of each individuals’ drug initiation by substance, injection initiation order and drug use duration. The analysis continued throughout the writing process as further questions arose requiring exploration.

## Results

### Ethnographic context

Huntington, the county seat of Kanawha, West Virginia, is a small urban center that retains strong connections to adjacent rural areas. While spending time with the participants in their daily lives, the ethnographic team found widespread involvement of women in sex work, with male sex work mentioned by men as an accepted source of income. Recent diagnosis with HIV was also common and openly acknowledged. Many of the interviewees were living homeless, whether in tents or abandoned houses.

Although the study location was a small city, people who used drugs frequently encountered each other face-to-face in a narrow geographical area of the city center. Despite this, there was often lack of trust between members. Most of the participants were either originally from the town or from nearby counties, with a few from adjoining states. Travel or migration from beyond these areas was unusual. Women frequently gave accounts of sexual violence and exploitation while men mentioned rapes of their mothers, girlfriends or acquaintances with whom they used drugs.

### Thematic findings

Our findings suggest that the form, timing and extent of drug consumption among participants was influenced by four stages of the changing drug supply, which in turn reflected back on demand. The widespread availability and consumption of pharmaceutical opioid pills in this location set in motion a series of steps that, for some, has logically led to the development of injection polypharmacy through the addition of stimulants to existing opioid injecting repertoires. Issues of economy, availability, increased social acceptability and the erosion of taboos and stigma all played their parts in the uptake of methamphetamine and opioid co-use by injection.

Among the 30 participants already injecting opioids, all had adopted MA use by injection, a less common mode of use for this substance. Participants reported using heroin from 3 to 23 years, injecting heroin 2–23 years and injecting methamphetamine from 4 months to 23 years. A striking finding was the distinct pattern of individual drug initiation that matched each progressive supply wave of the epidemic. Of the 28 participants who gave a clear account of their drug initiation sequence, 25 had initiated opioid use with pharmaceutical pills, followed by heroin, often mixed with or replaced by fentanyl and subsequently had added methamphetamine forming a ‘four wave initiation sequence’. We term this the ‘dominant sequence’.

Of these 25, eight reported some contemporaneous or previous use of cocaine powder or crack. Three of the 28 deviated from the dominant four wave initiation sequence, with one moving from methamphetamine to opioid pills to heroin, one from pills to crack cocaine prior to heroin and a third from opioid pills straight to co-use of meth and heroin. Some may have transitioned from opioid pills to heroin after fentanyl became a significant adulterant or replacement, producing a truncated ‘three wave initiation sequence’ while others clearly remembered using heroin when it was previously available without fentanyl. Most of the interviewees referred to ‘heroin’ as the substance they currently used rather than ‘fentanyl’ but often acknowledged the widespread presence of the latter in the opioid supply. This ambiguity can make it difficult to differentiate between intentional and unintentional transition to fentanyl.

Although the sample is small and non-representative, it is noteworthy that although the age of the participants ranged from 24 to 50 years we saw the same transition sequence, with no birth cohort effect. Most of the participants who reported transitioning from opioid pills to heroin/fentanyl did so between 2010 and 2015 during the second and third waves of the overdose epidemic, with uptake of methamphetamine following afterwards.

Modes of administration also followed a strong pattern. Of the 28 giving clear accounts, injection initiation occurred with an opioid in 25 respondents, whether pills (n = 9) or heroin/fentanyl (n = 16) rather than with methamphetamine. The remaining three had initiated injecting either with methamphetamine or crack cocaine. The few who mentioned any experience of smoking or snorting methamphetamine had done so in addition or prior to starting to inject it.

The significance of these patterns of drug progression and injection initiation reflect both changes in supply and in demand, with higher availability, lower price and altered attitudes to the new supply as more cost-effective, desirable or less stigmatized.

### Transitions to injecting and the transformation of stigma

Prior experiences with opioids proved critical to later adoption of methamphetamine by injection. Typically, several interviewees recalled their initial reluctance to inject and how this had been overcome with the economic and dependence-related demands of opioid use and the proximity of injecting norms among close associates. Jackson, a man in his 40s who had started injecting two decades earlier and followed the dominant drug initiation sequence (opioid pills-heroin-fentanyl-methamphetamine), had developed a high opioid tolerance when oxycodone (*OxyContin*) was easily available. He had obtained it from ‘pill mills’, clinics with lax medical standards providing prescriptions for large amounts of opioid medication often in return for cash payments. However, after such prescribing was curtailed he was left with a substantial and expensive habit and a limited supply, making injecting seem a more attractive option:

- *S:[…] I was probably using maybe as many as 10 Oxy 80’s a day. […] It was a huge habit. And at $80 a pill, so that was $800 a day. […]*
- *I:But you’re not paying that much. You’re at the wholesale level, yeah.*
- *S:Um, while the pill mill doctors were going on I was. But, uh, after they was gone I was definitely paying that much.[…]*
- *I:So go back to the point at which you started injecting then. Is it around the same time, uh, 800 mg a day habit or is it –*
- *S:It is that time.*
- *I:Who introduces you to injection?*
- *S:One of my friends that I knew introduced me […] and I was totally against injecting drugs, that’s the craziest thing, you know, I mean, these people were crazy to ever do that. And I was driving around in my car one day and I had a 20 mg* OxyContin *and I remember thinking, I crush this up and snort it it’s not gonna help me a bit. But my buddy’s over here and I’ll have him inject’em. I’m gonna go over there and have him show me.*

When Jackson and others added methamphetamine to their existing repertoire there was therefore little resistance to injecting it and several observed that once they had started injecting opioids that became their preferred route. Noah, a man in his 30s, started snorting oxycodone (*OxyContin*) in nearby Ohio and then transitioned to snorting heroin prior to the major wave of fentanyl adulteration. Heroin injecting followed, and then more recently methamphetamine:

- *I:[…] And when did you make the switch over to injecting?*
- *S:When I was probably 28. […]*
- *I:And then it was injecting everything from that point on or were there some things you would stay snorting?*
- *S:No it was inject everything even the experimental.*

Caleb, a man in his 40s, who followed the dominant initiation sequence, started injecting with heroin and described the typical resistance to injecting which he and others stigmatized:

*And [in West Virginia] there was a huge demand for pills and they would be snorting them, which then led to the, uh, shooting them up, the intravenous type of use, right. Um, of course, you know, a lot of us were like, oh, we’ll never go down that route, you know, there’s a lot of people who were like that. Uh, they would basically do anything besides use a needle.*

However, once this boundary had been crossed, there was no inhibition to injecting other drugs. Caleb’s strong attraction to the injecting experience transferred over to methamphetamine which he had taken up 4 months before the interview:

- *I: Why did you not smoke [meth]?*
- *S: Because it gave me more of a rush. I tried smoking it […] I already enjoyed the process of the needle so I continued with that.*

### The transformation of methamphetamine

Considering that methamphetamine was being produced within the state for at least a decade prior to the study, the question arises as to why it was not taken up earlier by this population. We found that transformations in methamphetamine manufacture and supply shifts contributed to its adoption among participants injecting opioids in terms of accessibility, price and social meaning. At the time of the research, two products were available: the locally produced ‘shake and bake’ and the new Mexican-sourced ‘ice’ or ‘cream’, which, when mixed with an opioid was called a ‘speedball’, a term commonly used elsewhere to refer to a cocaine-heroin mixture.

There was general agreement about ice’s lower price compared to the older product, sometimes even being given away for free or traded for other goods. This led some using opioids who did not favor the drug to accept it when offered. Liam, a man in his 30s whose drug initiation followed the dominant sequence, explains, “The truth is I don’t like ice. […] I do it often, I’ve never paid for it. I’ve never paid for it because ice especially around here is practically free. A week’s supply of ice costs about 40 bucks.” Allison, a woman in her 20s whose drug initiation also followed the dominant sequence, commented, “In shake ‘n’ bake you only get like a little bit for a certain amount [of money] and other ice you get more for the same amount.”

Discussions about methamphetamine revealed the drug’s material and symbolic transformations. To obtain shake’n’ bake, interviewees explained that personal connections to local producers were needed, impeding its use in the past, while ice was now easy to acquire in larger towns and cities. Chase, a man in his 30s, had been using opioids most of his life. Atypically, he had started using heroin before opioid pills and had latterly taken up ice, noting this significant change in availability and accessibility:

- *I:When did speed [methamphetamine] come into the picture for you?*
- *S:About like 4 years ago.*
- *I:And why do you think that change happened or was it not available before?*
- *S:It was available but you would’ve had to know people that make shake ‘n’ bake.*

Opinions varied as to how far ice had traveled from its place of origin, ranging from other West Virginian cities to other states and Mexico and revealed tensions between rural and urban identities. Among our respondents, all living in a small urban area, some stigmatized shake ‘n’ bake, associating it with undesirable “hillbilly” stereotypes of the rural population. Noah, noting the change in availability, expressed these attitudes, referencing the US horror movie series ‘Wrong Turn’ about a group of travelers attacked by cannibals when driving in an isolated part of rural West Virginia:

- *I:Do you think there’s anything that’s changed significantly with like the drug scene in the last five years?*
- *S:Oh yeah. The availability of both heroin and cream [methamphetamine]. Five years ago you wouldn’t even find – you’d probably search all day to find you methamphetamines and you’d probably actually have to go out here to Wayne County which is a hillbilly… way out where ‘Wrong Turn’ type stuff.*

Caleb explains the connection between meth’s availability and acceptability in the town, contrasting stigmatizing attitudes towards the domestic product with the now mainstream imported ice:

- *I:So tell me more about methamphetamine’s availability. So back when you were doing heroin, was meth not part of the scene?*
- *S:Correct. It wasn’t, or I’d heard it was but liken you know, it was seen like a dirty thing to do. It was something more like rednecks do, it wasn’t really part of the scene. It was kind of an outlier. A lot of the people who did it cooked it, did the shake ‘n’ bake kind of thing. It wasn’t trafficked in here like it is now.*

### Methamphetamine distribution

During informal conversations during the ethnography we heard about professional dealers visiting from Detroit but the local dealers were generally reported to be fellow consumers of heroin/fentanyl and/or methamphetamine. Observation of the participants pursuing drug transactions revealed that barter was a commonly accepted means of exchange for drugs in the town, suggesting the presence of non-professional user-dealers at the retail level. Two participants worked together to find someone willing to trade a quantity of heroin for meth while Cameron, a man in his 20s whose use had also followed the dominant sequence, reported bartering a range of goods desired by people using MA in exchange for the drug:

- *S:[…] So if I can, you know, I won’t spend cash money on meth because it’s just weird – people that are on meth, I mean, they get – there’s certain items that they – almost every one of em want.*
- *I:Like what?*
- *S:An example would be adult coloring books and markers. You know, they all – I guess something to focus on. […] So, but my group, it’d be like flashlights, bicycles, everybody wants a bicycle, flashlights […]*

While networks for shake ‘n’ bake distribution were more restricted than for the newer imported product, the high degree of ice diffusion in the town may have been assisted by the rising number of small-scale dealers who had started to sell heroin/fentanyl a few years earlier. This provided a ready distribution system for ice and other new drugs as they appeared and also meant that some dealers sold both heroin/fentanyl and methamphetamine, potentially facilitating consumers of opioids in developing their co-use. Noah, who had sold opioid pills earlier in his life in Ohio, explained how he now sold both heroin and meth as well as other drugs:

- *I:[…] Okay do you still sell drugs?*
- *S:Yeah.*
- *I:What kind?*
- *S:Those two [heroin and methamphetamine], the cream, well actually whatever I can get my hands on really.*

Owen was in his 20s and had started opioid pill use aged 11, then following the dominant sequence. He described a range of drug selling patterns among people using heroin and/or meth:

- *I:Do people sell both ice and heroin or is it different dealers?*
- *S:I mean you got some that do sell both and you then got some that are you know like they like heroin so they won’t sell it because they ain’t gonna benefit. You know what I mean? And you got some that love ice but hate food [heroin], don’t even want to touch it but they’ll sell it so they can get their ice.*
- *I:It sounds like a lot of people are using and selling.*
- *S:Yeah, almost every one of them is using, almost every single one of them.*

### Demand for other drugs limited by supply

Ice’s new dominant market position encouraged its uptake over other substances whose intrinsic effects were in some cases preferred, as with both cocaine and cannabis. Several respondents described how crack and cocaine powder had become less available in the area in recent years prior to the arrival of ice. Like other participants, Liam started his opioid use with pills and transitioned to injecting with heroin, then adding methamphetamine by injection. Cocaine had also featured in his drug repertoire but despite personal preference, local drug market conditions determined his switch to ice:

- *I:So when did you start using [methamphetamine] on top of the heroin?*
- *S:Well that was just in the past few years because my thing has always since I was young been cocaine […] I was using the cocaine to balance. I switched when I moved here primarily because there’s no good quality cocaine. […] It’s all garbage and it wears off way too quickly. It’s overpriced, it’s cut too bad. Ice, it lasts 3 times as long, it’s a quarter of the price and it’s constant, it’s everywhere you go.*

Others explained that they had used crack cocaine and later methamphetamine regardless of preference or enjoyment of the drug experience itself but as a counter to the sedative effects of opioid use. Prior to the influx of high potency Mexican-sourced ice in the area, cocaine had sometimes fulfilled this function. Sylvie, a woman in her 30s, had used cocaine prior to methamphetamine and explains this need:

*And the thing is, you know, even when, you know, you realize heroin is a downer, right? And so you have to have it and it makes you feel better but if I’ve got it after so long the shit puts you to sleep so you’ve gotta have something to keep you awake. […] So you know used to, we’d smoke crack. So I mean we was never just a heroin addict, we were fucking heroin junkies and crackheads.*

- *Q:If you have a choice between crack and ice which would you choose, like which is your preference?*
- *A:God, I really don’t fucking like either.*

Cameron was among those who expressed a preference for cannabis over heroin but found it harder to obtain in the town:

*I know 5 or 6 places I can get heroin. I don’t know where I can get a bag of weed right now […] I have to go through people, I have to search. I mean I can find it. That’s my true, that’s my number one. That was always my, if I’m not sick, that’s my choice of drug.*

Justine, a woman in her 30s whose drug use progressed in the dominant sequence and had also included crack cocaine, also preferred cannabis over heroin but was constrained by market conditions:

- *S:[…] If I could get the weed that would be my main choice […]*
- *I:Like if you had 20 bucks right now how fast could you get heroin?*
- *S:Just right up the corner.*
- *I:Right. How long until you find weed?*
- *S:Weed it would probably take almost all day. […] To be honest I can walk right across the street probably right now and get a pack [of heroin], you know, but if I want a joint it would probably take me all day just to find a joint.*

The addition of injected methamphetamine by existing consumers of opioids reflects transformations in the potency of the substance itself, its social profile and attendant stigma, its price and availability. While not inevitable, there is an internal logic to the adoption of methamphetamine by those using opioids and its consumption by injection rather than non-injection routes. The choice of stimulant between cocaine or methamphetamine partly reflected local market conditions, with price, availability and quality taking precedence over drug preference.

## Discussion

The sequence of drug initiation seen in this study sample was neither irresistible nor inevitable yet it is striking to see the progressive uptake of substances among almost all of the participants following the successive supply waves of the overdose epidemic *regardless of age*. The absence of a birth cohort effect on patterns of progression suggests that the shared external environment, acting across age groups, was more significant in choice of opioid or stimulant than individual ‘coming of age’ developmental influences. Starting their opioid use with prescription pills, moving to heroin and then heroin/fentanyl and finally adding methamphetamine, with injection initiation preceding methamphetamine, suggests a strong relationship between supply changes and drug and mode of use initiation.

Unlike Golub et al.’s ‘drug generations’ model, where the variation in drug use over time arises from changing drug preferences between birth cohorts, the influence of changes in supply on *multiple generations simultaneously* was more significant. Furthermore, these four connected waves of the epidemic originated not in a small subpopulation experimenting with risk-taking but with the mass distribution across generations of a prescribed or diverted supply in the form of pharmaceutically controlled opioids. This broad introduction of pharmaceutical opioids to vulnerable populations, followed by the widespread availability of heroin and fentanyl, proved critical in easing the path to injection initiation. Compared with other injectable drugs, heroin may have a faster path and a higher rate of progression to injection than MA [70–72], making heroin’s uptake particularly important in predicting injection drug use, although contrasting patterns have been found in different national contexts [73]. The subsequent ‘dominant sequence’ of drug initiation seen here, culminating in injected co-use of methamphetamine and opioids, can be traced back to this original starting point, so that the influence of initial prescription pills supplies had a much longer reach than previously thought.

However, despite the importance of supply influences shown here, availability alone does not cause use and demand for intoxicating substances must exist to some degree before they are consumed. Economic deprivation, adverse life events and cultural norms are important influences on the initiation and continuance of heroin and methamphetamine use and injecting [74–78]. The extreme trauma described by participants and the study setting suggest that these were likely significant factors for their involvement with these low price, high potency substances. The specific attractions of combining opioids and methamphetamine are examined in greater detail in a companion paper [41] but the *form, timing and extent* of this consumption was influenced by the changing drug supply, which in turn *reflected back* on demand. We can see this reflective relationship in market changes and the transformation of drug-related stigmas.

Participants who reported formerly stigmatizing injecting adopted it under the pressure of supply constraints and with the assistance of peers: limitations on the availability and affordability of opioid pills amidst their rising tolerance and dependence could transform injecting from an undesirable mode of use to an acceptable and ultimately preferred option, which in turn favored the uptake of other drugs by injection such as methamphetamine. The new supply of ice, associated regardless of accuracy, with urban rather than rural sellers, positively influenced demand among these participants. Unlike those in Cunningham et al.’s 2008 study, the dominance of Mexican-sourced methamphetamine did not result in its uptake by smoking among this particular population. When considering the generalizability of these findings, some stigmas, such as those around injecting drug use, are widely found but others may be limited to this particular context. The tensions between rural and urban identities which negatively characterized locally manufactured MA may not be broadly typical and this should be borne in mind in future research. Variations in drug distributions also need to be considered, with prescription opioids remaining available at higher levels and for longer in some states, including West Virginia, than others [79, 80].

The difficulty of reducing the supply of drugs such as heroin, cocaine and methamphetamine in the market without intensifying drug-related harm has been evident in the repeated attempts to do so but there remains the possibility of *increasing* the supply of less harmful drugs and so altering the balance of availability. Our findings suggest that for some, the higher risk drugs they were using were not their preferred choice. The reported relative shortage of cannabis, the favorite drug of some participants, may steer people away from non-injectable drug use towards opioids and methamphetamines.

West Virginia, with some legal access to medicinal cannabis but strict laws about non-medical possession, remains one of the 19 US states that have not decriminalized cannabis possession. Interventions to reduce demand for high risk substances and modes of use in West Virginia are urgently needed but there are also some possible supply side interventions that could reduce harm. Research findings suggest that laws allowing recreational cannabis use in the US reduce opioid mortality; the state of Colorado’s cannabis legalization was followed by a reversal of the upward trend in opioid overdose deaths [81, 82]. A national study found decreases of 20–35% in opioid related deaths following the introduction of recreational cannabis laws,

although concerns remain that weak regulation of the industry is allowing profit to be prioritized over public health [82, 83]. While its adverse health effects should not be overlooked, easier access to cannabis for recreational use on a tightly regulated, not-for-profit basis could divert potential and current consumers of injectable drugs of a higher dependence and overdose potential. The potential for cannabis as a substitute for opioid pain relief has attracted considerable attention; observational data exists regarding cannabis’ analgesic effects but rigorous clinical evaluation has been hampered by a lack of consistent formulations and randomized controlled trials [84, 85].

The sale of methamphetamine and opioids from the same sources may also create greater risk of co-use. A central rationale of the Dutch ‘separation of markets’ policy of the 1970s onwards [86, 87] is that buying cannabis in this way avoids contact with dealers who may also sell other drugs. The specific regulatory mechanisms by which legal access to cannabis is accomplished are critical. If we are to address both demand and supply for substances that are killing so many, we cannot expect, against all evidence, to foil the continuous ingenuity of free market suppliers. As we have seen in the opioid pharmaceutical and tobacco industries, profit spurs innovation but has proven a poor caretaker of public health [88, 89].

Alternative models of supply through state monopolies, as with alcohol, or nationalized health service programs with heroin and other opioids, could reduce harms as they lack the market incentives to expand consumption and can control product quality [90, 91]. A number of countries, including Canada and Switzerland, have prescribed opioids to dependent patients using varied models of medical prescription and contrasting goals, whether as a form of treatment, harm reduction or, in England during the late 1960s, to both treat individuals and control the wider drug supply [91–93]. They have shown proven effectiveness in improving individual health while their potential for attracting patients away from other suppliers can be influenced by the nature of the wider drug market, prescribed dose levels and the degree of autonomy afforded to participants [94, 95].

Previous studies have highlighted participant beliefs surrounding the ability of MA to prevent or reverse opioid overdose [35, 41] while mortality data showing rising numbers of deaths involving combinations of fentanyl and methamphetamine suggest the opposite [9]. A potential answer to this conundrum may lie in a mouse-model study which shows a dose-dependent relationship between fentanyl and methamphetamine, with amphetamines depressing respiration at lower doses, potentially increasing susceptibility to opioid overdose but increasing respiration at higher doses [96]. Further research into the applicability of these findings to humans is urgently needed and may provide the basis for harm reduction interventions for people who co-use methamphetamine and opioids.

As well as overdose concerns, co-use of MA and opioids raises issues regarding the efficacy of treatment for substance use disorders. A systematic review of the effects of methamphetamine on people seeking treatment for opioid use disorder found generally negative correlations between MA use and both receipt of and retention in treatment programs for opioid use disorder [97]. This poses an issue for substance use disorder progress in general, not only by worsening treatment outcomes for opioid use disorder but also due to the fact that there are currently no medication assisted treatment options for people who use methamphetamine. However, contingency management—a behavioral intervention that provides participants with incentives contingent upon testing negative for various substances—has proven an effective strategy for people using stimulants [98, 99], although narrow definitions of what constitutes success may inhibit participation [100].

While opioid injecting already raises the risk of blood borne disease transmission, the addition of methamphetamine may increase it further, whether through injection practices or high risk sexual behavior [32, 101, 102]. There is also some evidence that methamphetamine heightens physiological risk of HIV transmission during exposure [103]. The US, previously making progress towards reducing HIV transmission, has seen several injection-transmitted outbreaks, including in West Virginia.

The results of this study, while not necessarily representative of all drug markets, highlight the importance of supply in shaping the uptake and expansion of drug use and at the same time reflect the difficulties in suppressing drug production and supply when it is motivated by profit, particularly in an environment of economic deprivation. While the effects of controls on precursor chemicals have been varied and unpredictable, Mexican-based producers were able to successfully adapt their manufacturing process to meet demand and expand their market in the US. An inadvertent effect was to conjoin the methamphetamine and illicit opioid supply chains in a way previously unseen in the US and this may have assisted in the spread of co-use. In a similar way, earlier research found that heroin and cocaine, when both sourced from Colombia, proved to be a popular polydrug combination when sold together or near each other in the US city of Philadelphia [104].

Limitations from this study are those usually applicable to qualitative research. The sample may not be representative of the wider population of people using drugs. Sampling purposively selected those who used both methamphetamine and heroin/fentanyl, excluding those who did not take up methamphetamine on top of their existing opioid use, although this did not affect the order of uptake. While the research was a ‘snapshot’, condensed into one week, it was a repeat visit to the town after two years.

## Conclusion

This paper contributes to understanding macro-level trends in drug supply, use and harms and offers new insights at the level of individuals, helping to connect micro-level behavior patterns to the evolving macro-level context. It challenges existing models of drug epidemics and relations between methamphetamine sources and modes of use. In order to address the current fourth wave of the US overdose epidemic where stimulants and opioid co-use contribute heavily to mortality, it is essential to unpack its origins. Our findings of a clear pattern of drug initiation in West Virginia that align with the national supply waves of the overdose epidemic, interwoven with changes in drug-related stigma, show the importance of both specific cultural influences and wider changes in drug price, potency and availability. To be effective in addressing these challenges, harm reduction efforts need to address both supply and demand at the national and local levels.

## Data Availability

Data and materials are of a sensitive nature and are consequently not available to those outside of the study team. A US Federal Certificate of Confidentiality issued by the National Institutes of Health/National Institute on Drug Abuse protect the data and its collection.

## References

[1] Courtwright DT (2001). Forces of habit: drugs and the making of the modern world.

[2] Royal College of Psychiatrists in Drugs: Dilemmas and choices. 2000, Gaskell: London. p 185–213

[3] Fowler T (2007). Exploring the relationship between genetic and environmental influences on initiation and progression of substance use. Addiction.

[4] Verweij KJ (2010). Genetic and environmental influences on cannabis use initiation and problematic use: a meta-analysis of twin studies. Addiction.

[5] Edström B (2015). The forgotten success story: Japan and the methamphetamine problem.

[6] Parker H, Newcombe R, Bakx K (1987). The new heroin users: prevalence and characteristics in Wirral. Merseyside British Journal of Addiction.

[7] Jalal H (2018). Changing dynamics of the drug overdose epidemic in the United States from 1979 through 2016. Science.

[8] Centers for Disease Control and Prevention, N.C.f.I.P.a.C. *The Drug Overdose Epidemic: Behind the Numbers*. Opioids 2022 [cited 2022 08/26/22]; Available from: https://www.cdc.gov/opioids/data/index.html

[9] Hedegaard, H., et al., Drug overdose deaths in the United States*, 1999–2020.* NCHS data brief, 2022(428) 34978529

[10] Ciccarone D (2021). The rise of illicit fentanyls, stimulants and the fourth wave of the opioid overdose crisis. Curr Opin Psychiatry.

[11] Dasgupta N, Beletsky L, Ciccarone D (2018). Opioid crisis: no easy fix to its social and economic determinants. Am J Public Health.

[12] Monnat SM (2018). Factors associated with county-level differences in U.S. drug-related mortality rates. Am J Prev Med.

[13] Mars SG (2014). "Every 'never' I ever said came true": transitions from opioid pills to heroin injecting. Int J Drug Policy.

[14] Lankenau SE (2012). Initiation into prescription opioid misuse amongst young injection drug users. Int J Drug Policy.

[15] Cicero TJ, Ellis MS The prescription opioid epidemic: a review of qualitative studies on the progression from initial use to abuse. Dialogues in clinical neuroscience, 2022 10.31887/DCNS.2017.19.3/tcicero PMC5741109 29302223

[16] Ciccarone D (2019). The triple wave epidemic: Supply and demand drivers of the US opioid overdose crisis. Int J Drug Policy.

[17] Cicero TJ, Ellis MS, Surratt HL (2012). Effect of abuse-deterrent formulation of OxyContin. N Engl J Med.

[18] Harocopos A, Allen B, Paone D (2016). Circumstances and contexts of heroin initiation following non-medical opioid analgesic use in New York City. I J Drug Policy.

[19] Novak SP (2016). Initiation of heroin and prescription opioid pain relievers by birth cohort. Am J Public Health.

[20] Ciccarone D, Ondocsin J, Mars SG (2017). Heroin uncertainties: Exploring users' perceptions of fentanyl-adulterated and -substituted 'heroin'. Int J Drug Policy.

[21] Green TC, Gilbert M (2016). Counterfeit medications and fentanyl. JAMA Intern Med.

[22] Palamar JJ (2022). Trends in seizures of powders and pills containing illicit fentanyl in the United States, 2018 through 2021. Drug Alcohol Depend.

[23] Mars SG, Rosenblum D, Ciccarone D (2019). Illicit fentanyls in the opioid street market: desired or imposed?. Addiction.

[24] Carroll JJ (2017). Exposure to fentanyl-contaminated heroin and overdose risk among illicit opioid users in Rhode Island: a mixed methods study. I J Drug Policy.

[25] Kral AH (2021). Transition from injecting opioids to smoking fentanyl in San Francisco. California Drug Alcohol Depend.

[26] Daniulaityte R (2019). Street fentanyl use: Experiences, preferences, and concordance between self-reports and urine toxicology. Int J Drug Policy.

[27] Rothberg RL, Stith K (2018). Fentanyl: a whole New World?. J Law Med Ethics.

[28] Drug Enforcement Administration, National Drug Threat Assessment. 2019, Department of Justice

[29] US Drug Enforcement Administration, Fentanyl Flow to the United States,* DEA Intelligence Brief.* (2020)

[30] Wang C (2022). The evolving regulatory landscape for fentanyl: China, India, and global drug Governance. Int J Environ Res Public Health.

[31] U.S. Customs and Border Protection. Drug Seizure Statistics. 2024 02/13/2024 [cited 2024 February 22]; Available from: https://www.cbp.gov/newsroom/stats/drug-seizure-statistics

[32] Glick SN (2018). Increasing methamphetamine injection among non-MSM who inject drugs in King County. Washington Drug Alcohol Depend.

[33] Ellis MS, Kasper ZA, Cicero TJ (2018). Twin epidemics: the surging rise of methamphetamine use in chronic opioid users. Drug Alcohol Depend.

[34] Palamar JJ, Han BH, Keyes KM (2020). Trends in characteristics of individuals who use methamphetamine in the United States, 2015–2018. Drug Alcohol Depend.

[35] Daniulaityte R (2022). Lay knowledge and practices of methamphetamine use to manage opioid-related overdose risks. Int J Drug Policy.

[36] Silverstein SM (2021). “It’s crazy what meth can help you do”: lay beliefs, practices, and experiences of using methamphetamine to self-treat symptoms of opioid withdrawal. Subst Use Misuse.

[37] Lopez AM (2021). Co-use of methamphetamine and opioids among people in treatment in Oregon: a qualitative examination of interrelated structural, community, and individual-level factors. Int J Drug Policy.

[38] Duhart Clarke SE, Kral AH, Zibbell JE (2022). Consuming illicit opioids during a drug overdose epidemic: Illicit fentanyls, drug discernment, and the radical transformation of the illicit opioid market. Int J Drug Policy.

[39] Ivsins A (2022). The practice and embodiment of “goofballs”: a qualitative study exploring the co-injection of methamphetamines and opioids. Int J Drug Policy.

[40] Baker R (2021). "Like Yin and Yang": perceptions of methamphetamine benefits and consequences among people who use opioids in rural communities. J Addict Med.

[41] Ondocsin J, Holm N, Mars SG, Ciccarone D (2023). *The Motives and Methods of Methamphetamine and ‘Heroin’ Co-Use in West Virginia* Under peer review. Harm Reduct J.

[42] Flynn KC, Hoffer LD (2019). Transitioning illicit drug preferences and emerging user identities in Ohio: the proliferation of methamphetamine use among African Americans. J Ethn Subst Abuse.

[43] Ochoa KC (2005). Heroin overdose among young injection drug users in San Francisco. Drug Alcohol Depend.

[44] Suzuki J, El-Haddad S (2017). A review: fentanyl and non-pharmaceutical fentanyls. Drug Alcohol Depend.

[45] Drug Enforcement Administration, 2018 National Drug Threat Assessment, D.o. Justice, Editor. 2018

[46] Toske SG, McKibben TD (2022). Monitoring methamphetamine in the United States: a two-decade review as seen by the DEA methamphetamine profiling program. Drug Test Anal.

[47] Cunningham JK, Liu L-M, Callaghan R (2009). Impact of US and Canadian precursor regulation on methamphetamine purity in the United States. Addiction.

[48] Garriott W (2013). Methamphetamine in rural America: notes on Its emergence. Anthropology Now.

[49] Cunningham JK, Liu LM, Muramoto M (2008). Methamphetamine suppression and route of administration: precursor regulation impacts on snorting, smoking, swallowing and injecting. Addiction.

[50] US Drug Enforcement Administration, National Drug Threat Assessment Summary, D.o. Justice, Editor. 2016. p 80–81

[51] Drug Enforcement Administration, National Drug Threat Assessment. 2018, Department of Justice

[52] Strang J, Griffiths P, Gossop M (1997). Heroin in the United Kingdom: different forms, different origins, and the relationship to different routes of administration. Drug Alcohol Rev.

[53] Jones CM, Compton WM, Mustaquim D (2020). Patterns and characteristics of methamphetamine use among adults—United States, 2015–2018. Morb Mortal Wkly Rep.

[54] Ciccarone D, Bourgois P (2003). Explaining the geographical variation of HIV among injection drug users in the United States. Subst Use Misuse.

[55] Ciccarone D (2009). Heroin in brown, black and white: structural factors and medical consequences in the US heroin market. Int J Drug Policy.

[56] Kandel D, Faust R (1975). Sequence and stages in patterns of adolescent drug use. Arch Gen Psychiatry.

[57] Yamaguchi K, Kandel DB (1984). Patterns of drug use from adolescence to young adulthood: II. Sequences of progression. Am J Public Health.

[58] Morral AR, McCaffrey DF, Paddock SM (2002). Reassessing the marijuana gateway effect. Addiction.

[59] Van Gundy K, Rebellon CJ (2010). A life-course perspective on the “gateway hypothesis”. J Health Soc Behav.

[60] Golub A, Brownstein HH (2013). Drug generations in the 2000s: an analysis of arrestee data. Journal of Drug Issues.

[61] Scholl L (2019). Drug and opioid-involved overdose deaths—United States, 2013–2017. Morb Mortal Wkly Rep.

[62] West Virginia Department of Health and Human Resources, West Virginia drug overdose deaths historical overview 2001–2015. 2017: Charleston, WV. p 25

[63] Ondocsin J (2020). Hostility, compassion and role reversal in West Virginia’s long opioid overdose emergency. Harm Reduct J.

[64] Inciardi JA (2007). The diversion of prescription opioid analgesics. Law Enforc Exec Forum.

[65] O'Leary, S. Income Inequality at Historic High in West Virginia 2013; Available from: https://wvpolicy.org/income-inequality-at-historic-high-in-wv/

[66] Mars SG (2022). ‘The High Five Club’: social relations and perspectives on HIV-Related Stigma During an HIV Outbreak in West Virginia. Cult Med Psychiatry.

[67] Fessel JN, et al, Into the Epistemic Void: Using Rapid Assessment to Investigate the Opioid Crisis. Inside Ethnography: Researchers Reflect on the Challenges of Reaching Hidden Populations, 2019: p 120

[68] Christopoulos K, Olender S, Lopez AM, Lekas H-M, Jaiswal J, Mellman W, Geng E, Koester KA (2015). Retained in HIV care but not on antiretroviral treatment: a qualitative patient-provider dyadic study. PLoS Med.

[69] Glaser BG, Strauss AL (2017). Discovery of grounded theory: Strategies for qualitative research.

[70] Bluthenthal RN (2015). Factors associated with being asked to initiate someone into injection drug use. Drug Alcohol Depend.

[71] Bluthenthal RN, Wenger LD, Bourgois P, Valente T, Kral AH (2018). Differences in time to injection onset by drug in California: implications for the emerging heroin epidemic. Drug Alcohol Depend.

[72] Novak SP, Kral AH (2011). Comparing injection and non-injection routes of administration for heroin, methamphetamine, and cocaine users in the United States. J Addict Dis.

[73] Crofts N (1996). The first hit: circumstances surrounding initiation into injecting. Addiction.

[74] Svendsen K (2014). Persistent opioid use and socio-economic factors: A population-based study in N orway. Acta Anaesthesiol Scand.

[75] Williams CT, Latkin CA (2007). Neighborhood socioeconomic status, personal network attributes, and use of heroin and cocaine. Am J Prev Med.

[76] Payne J (2007). Women drug users in North Cumbria: what influences initiation into heroin in this non-urban setting?. Sociol Health Illn.

[77] Guise A (2017). The experience of initiating injection drug use and its social context: a qualitative systematic review and thematic synthesis. Addiction.

[78] Hobkirk AL (2016). A qualitative study of methamphetamine initiation in Cape Town, South Africa. Int J Drug Policy.

[79] Guy GP (2017). Vital signs: changes in opioid prescribing in the United States, 2006–2015. MMWR Morb Mortal Wkly Rep.

[80] Guy GP (2019). County-level opioid prescribing in the United States, 2015 and 2017. JAMA Intern Med.

[81] Livingston MD (2017). Recreational cannabis legalization and opioid-related deaths in colorado, 2000–2015. Am J Public Health.

[82] Chan NW, Burkhardt J, Flyr M (2020). The effects of recreational marijuana legalization and dispensing on opioid mortality. Econ Inq.

[83] Hall W (2018). The future of the international drug control system and national drug prohibitions. Addiction.

[84] Humphreys K, Saitz R (2019). Should physicians recommend replacing opioids with cannabis?. JAMA.

[85] Eichorn NL (2022). Making a joint decision: Cannabis as a potential substitute for opioids in obstetrics and gynecology. Best Pract Res Clin Obstetr Gynaecol.

[86] Wouters M, Korf DJ (2009). Access to licensed cannabis supply and the separation of markets policy in the Netherlands. J Drug Issues.

[87] MacCoun R, Reuter P (2001). Evaluating alternative cannabis regimes. Br J Psychiatry.

[88] Bates C, Rowell A, Tobacco Explained... The truth about the tobacco industry... in its own words. 2004

[89] Van Zee A (2009). The promotion and marketing of oxycontin: commercial triumph, public health tragedy. Am J Public Health.

[90] Room R, Örnberg JC (2019). Government monopoly as an instrument for public health and welfare: lessons for cannabis from experience with alcohol monopolies. Int J Drug Policy.

[91] Karamouzian M (2023). Challenges of implementing safer supply programs in Canada during the COVID-19 pandemic: a qualitative analysis. Int J Drug Policy.

[92] Stimson GV, Oppenheimer E (1982). Heroin addiction: Treatment and control in Britain.

[93] Uchtenhagen A (1997). Programme for a medical prescription of narcoticsa synthesis of results. Eur Addict Res.

[94] McNeil R (2022). Implementation of safe supply alternatives during intersecting COVID-19 and overdose health emergencies in British Columbia, Canada, 2021. Am J Public Health.

[95] Mars SG, The Politics of Addiction. Medical Conflict and Drug Dependence in England since the 1960s. First published, 2012 ed. Science, Technology and Medicine in Modern History, ed. J. Pickstone. 2021 (paperback edition), Houndsmill and New York: Springer

[96] Elder HJ (2023). Amphetamines modulate fentanyl-depressed respiration in a bidirectional manner. Drug Alcohol Depend.

[97] Frost MC (2021). The impact of methamphetamine/amphetamine use on receipt and outcomes of medications for opioid use disorder: a systematic review. Addict Sci Clin Pract.

[98] Brown HD, DeFulio A (2020). Contingency management for the treatment of methamphetamine use disorder: a systematic review. Drug Alcohol Depend.

[99] De Crescenzo F (2018). Comparative efficacy and acceptability of psychosocial interventions for individuals with cocaine and amphetamine addiction: A systematic review and network meta-analysis. PLoS Med.

[100] Clay S et al. Perspectives and sentiments on contingency management from people who use methamphetamine. Drug and Alcohol Review, 2023 10.1111/dar.13691 37248676

[101] Patterson TL (2005). Methamphetamine-using HIV-positive men who have sex with men: correlates of polydrug use. J Urban Health Bull New York Academy Med.

[102] Spindler HH (2007). Viagra, methamphetamine, and HIV risk: results from a probability sample of MSM. San Francisco Sexually transmitted diseases.

[103] Fulcher JA (2018). Brief report: recent methamphetamine use is associated with increased rectal mucosal inflammatory cytokines, regardless of HIV-1 serostatus. J Acquir Immune Deficiency Syndromes.

[104] Mars SG (2016). The textures of heroin: User perspectives on “black tar” and powder heroin in two US cities. J Psychoactive Drugs.

